# Iron-Deficiency Anemia as a Rare Cause of Cerebral Venous Thrombosis and Pulmonary Embolism

**DOI:** 10.1155/2012/497814

**Published:** 2012-05-31

**Authors:** Nicolas Nicastro, Armin Schnider, Béatrice Leemann

**Affiliations:** Division of Neurorehabilitation, Department of Clinical Neurosciences, Geneva University Hospital (HUG), 1206 Genève, Switzerland

## Abstract

Cerebral venous thrombosis (CVT) is a relatively rare cause of stroke and has a wide spectrum of unspecific symptoms, which may delay diagnosis. There are many etiologies, including hematological disorders, trauma, infection, and dehydration. Iron-deficiency anemia (IDA) has been reported as an extremely rare cause of CVT, especially in adults.

## 1. Introduction

Cerebral venous thrombosis (CVT) and pulmonary embolism (PE) share many risk factors such as hematological disorder, contraceptive use, tumor, dehydration, and trauma [1]. Iron-deficiency anemia (IDA) has been described as a cause of several adult cases of CVT the last few years [2, 3].

## 2. Case Report

**Figure 1:**
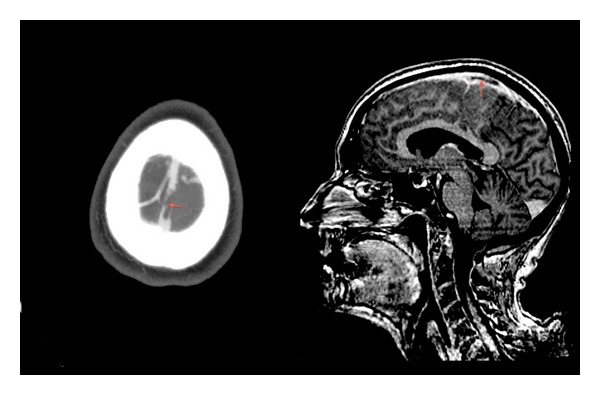
Brain computed tomography showing superior sagittal sinus thrombosis and implication of a cortical vein (arrows).

**Figure 2:**
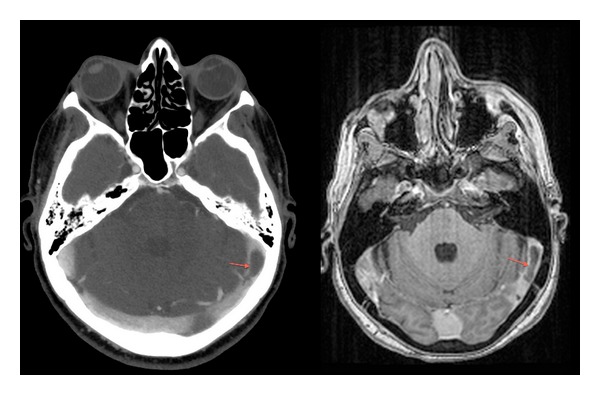
T1-weighted brain magnetic resonance showing left transverse sinus thrombosis (arrows).

**Figure 3:**
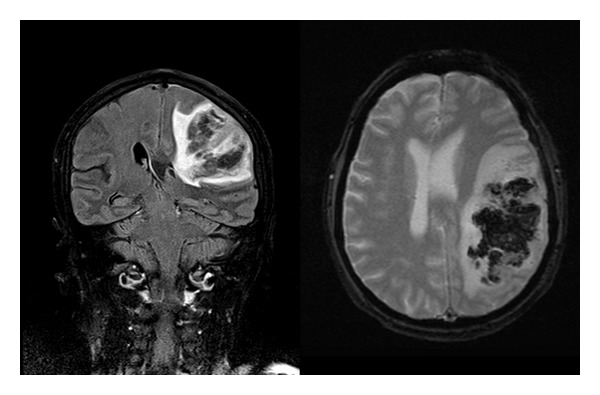
T2-weighted FLAIR magnetic resonance showing massive left frontotemporal hemorrhagic infarction.

**Figure 4:**
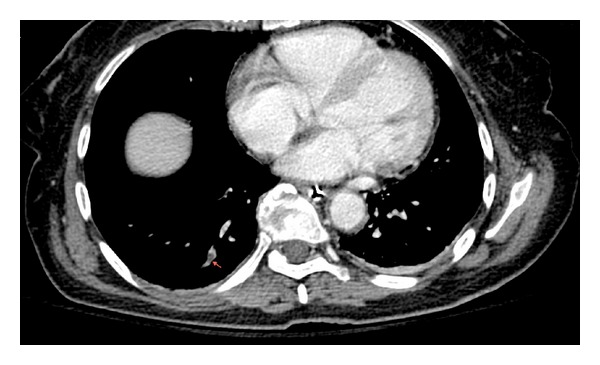
Computed tomography showing right subsegmentary pulmonary embolism (arrows).

A 63-year-old woman with no past history of coagulation abnormality, recent trauma, or hormonal substitution experienced sudden onset of headache followed by installation of right hemiplegia and global aphasia. Computed tomography and subsequent brain MRI revealed massive left frontotemporal hemorrhagic infarction and thrombosis of the superior sagittal sinus, left sigmoid/transverse sinus, and cortical vein (Figures 1, 2, and 3). Subsequently, chest tomography showed bilateral subsegmentary pulmonary embolism (Figure 4). Doppler did not reveal any deep venous thrombosis of the lower limbs.

The laboratory data showed severe hypochromic microcytic anemia with hemoglobin value of 3,4 g/L (normal range: 12–16). Serum iron concentration was 1 *μ*mol/L (*N *= 5–30), and ferritin concentration was 2 mg/dL (*N *= 11–137). No B12 vitamin or folate deficiency was found. Screening for coagulopathy was normal, including factor II, factor V (Leyden), activated protein C resistance, and antiphospholipid antibodies. Protein C, protein S, and antithrombin III were not interpretable because of early vitamin K antagonists therapy. Protein immunofixation electrophoresis demonstrated no gammopathy. Upper gastrointestinal and lower digestive endoscopies, mammography, endovaginal echography, thoracoabdominal and pelvic computed tomography were performed and failed to detect any malignant disease, source of active bleeding, or pelvic vein thrombosis.

The patient was treated by blood transfusion and anticoagulation with intravenous heparin, followed by vitamin K antagonists (acenocoumarol) for a total duration of six months. The brain MRI performed one month after the onset showed an almost complete repermeabilisation of the cerebral venous sinuses.

The patient's right hemiparesis gradually improved, as well as her global aphasia. The patient was discharged three months after the onset. The anemia gradually improved with a hemoglobin value of 13,0 g/L. Our assumption regarding the origin of the anemia was a vegetarian diet without a proper iron substitution.

## 3. Discussion

This case shows simultaneous thrombosis of multiple cerebral venous sinuses and pulmonary embolism associated with iron-deficiency anemia. As stated by Diaz et al. [4], concomitant pulmonary embolism was found in 11% of 203 cases of intracranial venous thrombosis. Several mechanisms have been proposed to explain the association between IDA and thrombosis, as iron is an important regulator of thrombopoiesis [5]: low iron levels disinhibit megakaryocyte activity [6], which provokes secondary thrombocytosis, thus leading to a hypercoagulable state [7]. In addition, microcytosis alters red cells deformability, which increases viscosity and possibly the risk of venous thrombosis [8]. Finally, anemic hypoxia secondary to iron deficiency may occur as the oxygen-carrying capacity of erythrocytes decreases, especially in situations where the metabolic demands are increased. All these conditions lead to a turbulent blood flow, causing platelets to come more frequently in contact with the endothelial lining [9].

In their prospective study of 121 patients with iron-deficiency anemia and cerebral venous thrombosis [10], Stolz et al. suggest a significant association of severe anemia (Hb < 90 g/L) and CVT. Our paper seems to support the fact that anemia may play a consistent role in the development of CVT. Concomitant pulmonary embolism could be explained by a local arterial thrombus, possibly also induced by anemia. Another hypothesis would be the embolization of a cerebral vein clot to the pulmonary arteries, as mentioned by Diaz et al. [4].
